# Assessing the Effects of Continuous Theta Burst Stimulation Over the Dorsolateral Prefrontal Cortex on Human Cognition: A Systematic Review

**DOI:** 10.3389/fnint.2020.00035

**Published:** 2020-08-04

**Authors:** Ronald Ngetich, Jing Zhou, Junjun Zhang, Zhenlan Jin, Ling Li

**Affiliations:** Key Laboratory for Neuroinformation of Ministry of Education, School of Life Science and Technology, University of Electronic Science and Technology of China, Chengdu, China

**Keywords:** dorsolateral prefrontal cortex (DLPFC), theta burst stimulation (TBS), cognition, pharmacotherapy, systematic review

## Abstract

Theta burst stimulation is increasingly growing in popularity as a non-invasive method of moderating corticospinal networks. Theta burst stimulation uses gamma frequency trains applied at the rhythm of theta, thus, mimicking theta–gamma coupling involved in cognitive processes. The dorsolateral prefrontal cortex has been found to play a crucial role in numerous cognitive processes. Here, we include 25 studies for review to determine the cognitive effects of continuous theta burst stimulation over the dorsolateral prefrontal cortex; 20 of these studies are healthy participant and five are patient (pharmacotherapy-resistant depression) studies. Due to the heterogeneous nature of the included studies, only a descriptive approach is used and meta-analytics ruled out. The cognitive effect is measured on various cognitive domains: attention, working memory, planning, language, decision making, executive function, and inhibitory and cognitive control. We conclude that continuous theta burst stimulation over the dorsolateral prefrontal cortex mainly inhibits cognitive performance. However, in some instances, it can lead to improved performance by inhibiting the effect of distractors or other competing irrelevant cognitive processes. To be precise, continuous theta burst stimulation over the right dorsolateral prefrontal cortex impaired attention, inhibitory control, planning, and goal-directed behavior in decision making but also improved decision making by reducing impulsivity. Conversely, continuous theta burst stimulation over the left dorsolateral prefrontal cortex impaired executive function, working, auditory feedback regulation, and cognitive control but accelerated the planning, decision-making process. These findings constitute a useful contribution to the literature on the cognitive effects of continuous theta burst stimulation over the dorsolateral prefrontal cortex.

## Introduction

Transcranial magnetic stimulation (TMS) is a method that non-invasively facilitates neural modulation of the specific targeted cortical brain areas and, therefore, makes it possible to examine their functional roles (Guse et al., 2010; Kaminski et al., 2011; Chung et al., 2018b). Essentially, TMS uses an electromagnetic coil to deliver electrical stimulation on the neural cortex via the scalp. While single pulse TMS may not last beyond the stimulation duration, another form of TMS called repetitive TMS (rTMS) induces longer-lasting plasticity that persists several minutes after stimulation (Klomjai et al., 2015). In addition to its relevance in brain research, rTMS has also been used for therapeutic purposes and neuroplasticity (Hwang et al., 2010; Kim et al., 2012; Lowe et al., 2018).

Previous studies suggest that rTMS may inhibit or excite the neural cortex. Actually, rTMS consists of the application of rhythmic trains of multiple TMS pulses (Bolognini and Ro, 2010), which may be of either lower or higher frequency. The lower frequency rTMS (<1 Hz) has an inhibitory effect, whereas the higher frequency rTMS (>1 Hz) accentuates the facilitatory effect (Fitzgerald et al., 2006; Caparelli et al., 2012). Moreover, Bolognini and Ro (2010) suggest that higher frequency rTMS is typically applied as single short trains having different intertrain intervals, and lower frequency rTMS is applied for a longer duration as continuous stimulation. It has also been established that the rTMS stimulation effects can extend to the distant interlinked cortical regions and are not entirely restricted to the stimulated site (Guse et al., 2010). This suggests that the possible cognitive effects after stimulation could be partly due to the secondary rTMS effects.

Over time, a new TMS protocol, theta burst stimulation (TBS), has emerged. According to Huang et al. (2005), TBS uses gamma frequency trains applied at theta rhythm (which mimics coupling involved in human cognitive processes, such as working memory). In addition, Cho et al. (2010) and Huang et al. (2005) distinguish the two types of TBS: intermittent TBS (iTBS), which they described as facilitatory, and inhibitory TBS, continuous TBS (cTBS). More so, recent studies have demonstrated the varied effects of cTBS and iTBS on different brain areas with cTBS causing more stable cognitive behavioral effects compared to iTBS, for which some recent studies have failed to establish its behavioral enhancement (Chung et al., 2018a; Hill et al., 2018).

Indeed, TBS has been found to have the same cortical modulatory effect as rTMS but with a fewer number of pulses. Ordinary rTMS needs between 20 and 30 min of stimulation to yield its full effect, which makes it relatively unfavorable in both treatment and experiment, whereas TBS proves to be more efficient by requiring only between 20 s and 3 min to be fully effective (Lowe et al., 2018).

Despite the fact that a significant number of TBS studies have focused on the motor cortex, a large majority of therapeutic and experimental research based on psychiatric disorders, such as depression and schizophrenia, and behavioral episodes, including addiction, have crucially concentrated on the prefrontal cortex (PFC). This is possibly due to its connection to vast features of cognitive operations (Postle and Rypma, 2000; Kubota et al., 2011; Engle, 2014). The current systematic review, therefore, focuses on the dorsolateral prefrontal cortex (DLPFC), a subregion of the PFC, with a theoretically connected test of cognitive performance as a product.

To be precise, we measured the cognitive effects of TBS stimulation over the DLPFC on the following domains: attention, a cognitive-behavioral process of selective concentration on a specific feature of information and its integration (Yantis, 2009; Bisley and Goldberg, 2013); working memory (WM), a process that enables brief storage and manipulation of cognitive information (Engle, 2014); planning, the identification and selection of the appropriate order of actions prior to actual performance (Kaller et al., 2011); decision making, a process of picking a preferred option from a range of alternatives (Georgiev et al., 2016); cognitive and inhibitory control, the capacity to undertake goal-oriented behaviors and the suppression of prepotent responses, respectively (Goghari and MacDonald, 2009; Diamond, 2013; Schmitt et al., 2016); language, an important cognitive process (Axelrod et al., 2015) and a fundamental element of human thought (Baars and Gage, 2010) that forms the basis of human communication and interaction with the immediate environment (Peeters et al., 2017); and broadly, executive function (EF), a generic term that incorporates a number of cognitive processes, such as the aforementioned domains (McKenna et al., 2017).

It is apparent that the above cognitive faculties play a key and interdependent role in humans' daily activities, ranging from simple tasks, such as remembering where you left your keys, to more complex tasks, such as problem solving and multitasking. Therefore, it is probable that an impediment to any of the cognitive functions may lead to mental health problems. In the present study, we systematically review studies that have investigated the effects of cTBS over the DLPFC on cognitive processes in healthy participants. We also include a few intermittent iTBS studies for comparison purposes. In addition, we review the application of cTBS and iTBS over the DLPFC in the treatment of pharmacology refractory depression to get insight into the usefulness and the uniqueness of TBS techniques in psychiatric intervention. Finally, we highlight the fundamental factors that can affect TBS experiments' outcome. We suggest that lack of neuronavigation in locating the stimulation target, use of suboptimal stimulation intensities, and lack of blinding (either single or double) can negatively affect the experimental results.

### Aim of the Study

Currently, high-frequency rTMS remains a frequently used paradigm to study the effects of rTMS on cognition (Preston et al., 2010; Lage et al., 2016), perhaps because it is a more popular paradigm, especially in depression. A systematic review by Guse et al. (2010) reports that most studies using higher frequency rTMS stimulations did not find significant cognitive effects. However, they noted a trend toward selective cognitive enhancement in a significant number of studies after high-frequency rTMS stimulation.

Importantly, despite having not received adequate popularity, lower frequency rTMS has proven to be safe and effective as an option for treating treatment-resistant depression (TRD) (Pallanti et al., 2012) and also in the treatment of psychotic disorders, such as schizophrenia (Stanford et al., 2008). Although a review on lower frequency rTMS did not find a significant conclusion to draw, it pointed out key areas of improvement (Lage et al., 2016).

Moreover, the continued progress in non-invasive brain stimulation research has led to the development of a new r-TMS protocol, TBS, which consist of iTBS and cTBS. To be precise, iTBS is facilitatory just like higher frequency, whereas cTBS, in the same way as lower frequency rTMS, is inhibitory. However, iTBS and cTBS take a shorter time of application to be effective, making it safer and more acceptable to participants and patients (Suppa et al., 2016). Intriguingly, despite the fact that cTBS applied especially over the DLPFC is now commonly used in the treatment of mental health disorders, such as depression, no review has been done on its cognitive effects when applied over this brain area. Actually, the fact that mental disorders are associated with deterioration in cognitive functioning and, consequently, their treatment aims at improving cognitive faculties makes such a review timely and necessary. Therefore, the present study systematically reviews the cognitive effects cTBS applied over the DLPFC on both patients and healthy participants. The included studies of iTBS over the DLPFC have been used to draw clear comparisons with its cTBS counterpart and, thus, provide more insights into the nature and the direction of cTBS effects and also to explore the effects of the combination of the two stimulations (cTBS and iTBS) particularly in the treatment of medication-resistant depression.

## Methods

### Selection of Studies

To effectively conduct our review, we adopted the methodologies used by previous similar systematic review studies, including Guse et al. (2010) and Lage et al. (2016). In addition, literature was systematically searched and reported in line with the Preferred Reporting Items for Systematic Reviews and Meta-Analyses.

A systematic literature search was conducted in the PubMed, PsycINFO, and Medline websites for studies published/accepted for publication in the period between January 1, 2008, and April 10, 2020. The major search terms used in the study included “cognitive effects of TBS,” “DLPFC cTBS effects,” “TMS protocol,” “cognitive control,” “executive function,” “rTMS effects over the PFC,” “non-invasive methods of brain stimulation,” “neural stimulation,” and “TBS in treatment of medication-resistant depression.”

**Figure 1:**
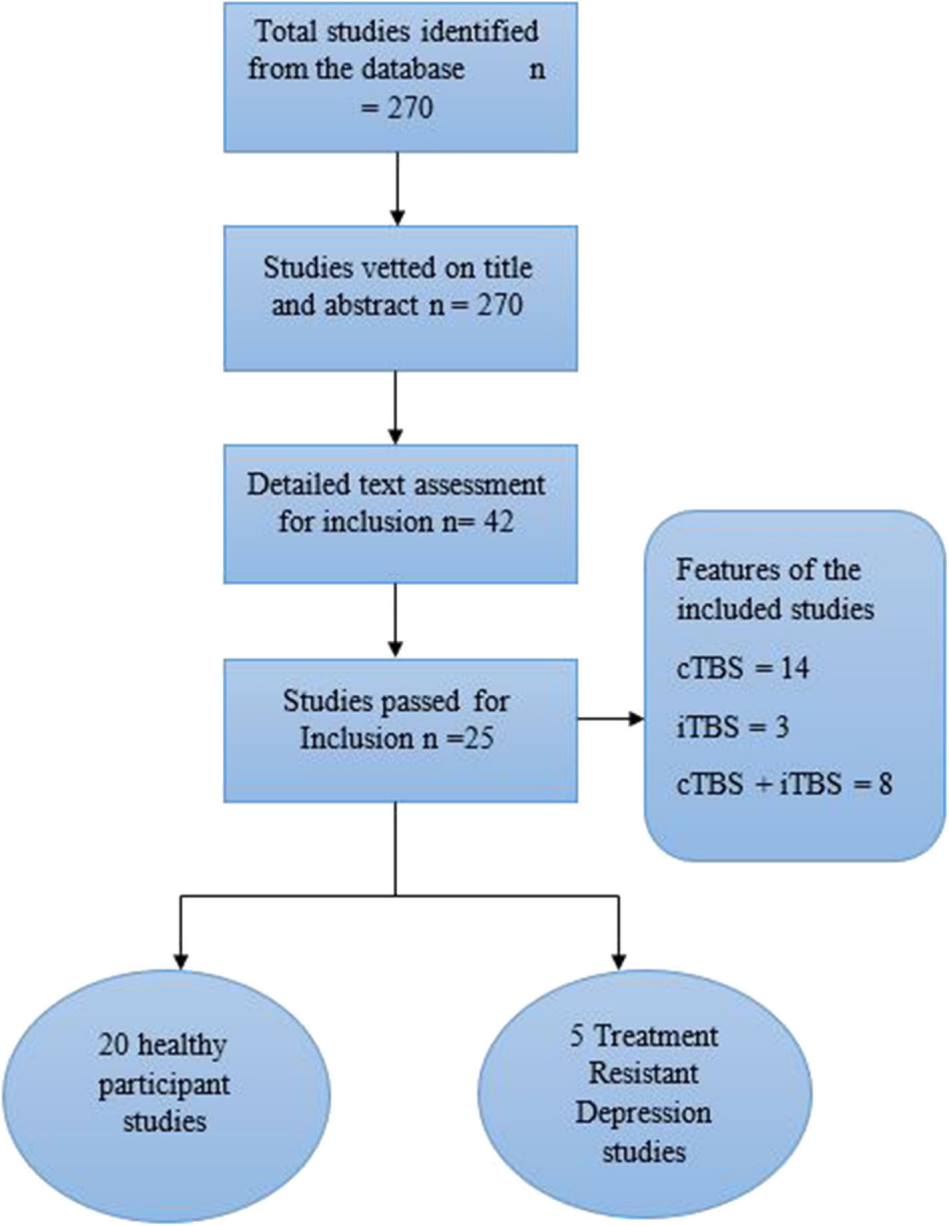
PRISMA flow diagram of systematic selection of studies.

From the search, 270 relevant studies were identified based on their titles and the evaluation of the study abstract. Out of the 270 studies, 100 were based on transcranial direct current stimulation and 72 on single-pulse TMS and rTMS, all of which were excluded. The remaining 98 TBS studies were vetted based on the specific brain regions they reported, and 42 papers involving the PFC were selected. The search was then narrowed down to 25 (20 healthy participants and five patient) main studies specific to the DLPFC, which were eventually included in the study (Figure 1).

### Assessment of the Risk of Selection Bias

Evidently, it is possible to have biases and inconsistencies when developing important questions that shape the scope of the systematic review (Ahmed and Sutton, 2012; Drucker et al., 2016). More so, it is possible that biases, such as publication bias, can arise during the process of selecting studies for inclusion (Drucker et al., 2016). Therefore, to ensure objectivity in selection, analysis, and interpretation of the available literature, the researcher must be cognizant of the selection biases and mitigate against them. In this regard, we assessed the validity of the included studies using Cochrane collaboration's bias assessment software (Shuster, 2011).

## Results

Generally, the heterogeneous nature of the techniques used to measure cognition, the number of participants, the specific cognitive domain in each study, the TBS parameters, and the sham/control experiments in the various studies included for analysis rendered the meta-analytical method an impracticable option. Therefore, a rigorous descriptive analysis was deemed the better option in the present review.

The main elements included in our analysis are cognitive effects of TBS stimulation, the motor threshold, the number of pulses *per session*, and the cognitive domains, such as working memory and executive function. The studies were categorized into either healthy participant or patient studies. The patient studies comprised only medication-resistant major depressive disorder (MDD). The specific elements were clearly tabulated for efficient analysis and precise understanding.

### Illustration of the Studies

The studies selected in this review had different features. Specifically, *n* = 7 adopted mixed experimental design, *n* = 5 used between-subject design, and more than 50% of the studies *n* = 13 are within subject (Table A1). In addition, the studies are classified as not blinded, single blinded, or double blinded. Among the studies included, *n* = 2 are not blinded, whereas *n* = 14 and *n* = 6 are single- and double-blind studies, respectively (Table A1). Notably, nearly all the selected patient studies, *n* = 4 out of 5, used both a mixed experimental design and double blinding, but the healthy participant studies had more varied features between them.

### Effects of TBS Stimulation on Different Cognitive Domains

We reviewed studies with different cognitive domains with some studies investigating more than one cognitive domain. Specifically, the major domains we reviewed are decision making, *n* = 6; planning, *n* = 1; learning, *n* = 1; working memory, *n* = 6; attention, *n* = 1; cognitive control, *n* = 1; inhibitory control, *n* = 1; language, *n* = 2; and executive function, *n* = 4 (one patient study) (Tables A2, A3). However, *n* = 4 patient studies did not measure specific cognitive domains (Table A3).

Additionally, we found that the DLPFC stimulation effect is dependent on the cognitive domain being investigated, the type of stimulation (iTBS or cTBS), and the specific hemisphere stimulated. The stimulation effect on each domain is described below.

#### Executive Function

The cTBS stimulation to the left and not to the right DLPFC in *n* = 2 studies impaired executive function performance (Ko et al., 2008; Lowe et al., 2018), whereas in *n* = 1, neither cTBS or iTBS to the left DLPFC had a significant effect on EF (Viejo-sobera et al., 2017). Intriguingly, in one patient study, the cTBS to the right seemed to worsen EF, a trend not noticed in the aforementioned healthy participant studies (Li et al., 2014).

#### Working Memory

For working memory, we establish that mixed results are reported in different studies. For instance, *n* = 3 WM studies used cTBS, and one study reports a significant decrease in WM performance after cTBS stimulation over the left DLPFC in a medium-load task while another one reports a slight inhibitory effect after cTBS over both the left and right DLPFC (Schicktanz et al., 2015; Vékony et al., 2018). On the other hand, one study did not find a cTBS stimulation effect over the left DLPFC (Viejo-sobera et al., 2017). Moreover, in the iTBS category, *n* = 2 studies report improvement in performance after iTBS over the left DLPFC (Hoy et al., 2015; Chung et al., 2018b); however, Chung et al. (2018b) report a stimulation effect only in a three-back task using 75% rMT. Alternately, *n* = 3 studies report no iTBS stimulation effect, two of which investigated iTBS over the left DLPFC (Viejo-sobera et al., 2017; Chung et al., 2018a), and one investigated iTBS over both left and right DLPFC (Vékony et al., 2018).

#### Decision Making

In decision making, it is interesting to note that TBS stimulation over the DLPFC yielded mixed results, which might lead to different interpretations on how such stimulation protocol can be used in the treatment of mental health disorders associated with impaired decision making. For instance, in *n* = 2 studies, cTBS over the right DLPFC decreased impulsive decision making while in one of these studies, iTBS was also applied over the right DLPFC but did not affect impulsivity (Cho et al., 2010; Soo et al., 2012). In addition, the cTBS over the right DLPFC impaired overall goal-directed behavior (GDB) while that over the left DLPFC only impaired GDB in low-WM participants (Smittenaar et al., 2013). Additionally, *n* = 1 study reports impaired decision making in an easy trial after cTBS over the right DLPFC (Georgiev et al., 2016). Furthermore, cTBS over the right DLPFC also impaired forgiveness decision making and increased revenge behavior in *n* = 1 studies (Maier et al., 2018). It is also interesting that in one study using Fish Game task, cTBS over right DLPFC did not affect decision making pertaining future events (Langenbach et al., 2019).

#### Planning, Attention, Learning, Language, and Cognitive and Inhibitory Control

Finally, varied effects on planning, learning, attention, language, cognitive control, and inhibitory control was also realized after TBS (iTBS or cTBS) over either the left or right DLPFC. In planning, cTBS to the right slowed down planning speed, whereas that to the left DLPFC accelerated the planning speed (Kaller et al., 2011). More so, the cTBS to the right DLPFC affected learning by enhancing reward sensitivity, and that to the left DLPFC increased avoidance-based behavior (Kaminski et al., 2011). Furthermore, the cTBS to the right DLPFC also reduced attention-dependent regulation of somatosensory event-related potential (ERP) (Bolton and Staines, 2011). On the language domain, one study reports that neither cTBS nor iTBS over the left DLPFC influenced language switching (LS) control (Pestalozzi et al., 2020). However, in one study, cTBS over the left DLPFC led to increased vocal compensation for pitch perturbations, which was electro-physiologically accompanied by a reduction in P2 cortical responses (Liu et al., 2020). These results on the effects of TBS over DLPFC on language regulation attract diverse arguments, where on the one hand, DLPFC does not regulate language switching, and on the other, it exerts a top-down control over voice production. Last, the cTBS to the right DLPFC reduced both inhibitory and cognitive control (Maier et al., 2018; Mcneill et al., 2018).

### Experiment Controls and Neuronavigation

In the present systematic review, *n* = 23 studies used either sham or vertex control to regulate the experimental confounds. Specifically, *n* = 18 studies used different sham stimulations, *n* = 4 studies used vertex stimulation, and one study used both sham and vertex, whereas *n* = 2 studies used neither sham nor vertex controls (Tables A2, A3). The sham stimulation designs ranged from the use of a sham coil *n* = 3, current directed up and outward, and stimulator output reduced to 6%, *n* = 1; active to passive placebo, *n* = 1; 45° coil rotation from the target, *n* = 2, and the majority of studies tilted the coil 90° to the target, *n* = 11, and *n* = 1 did not specify the adopted sham design.

Apart from the use of sham and vertex controls, researchers using TMS to explore neural modulation have come to appreciate the role of neuronavigation in accurately locating stimulation coordinates (Lage et al., 2016). In the reviewed studies, neuronavigation was adopted by only *n* = 11 out of *n* = 25.

### Motor Threshold and Pulses *per session*

#### Healthy Participant Studies

The present study reviewed *n* = 20 healthy participant and *n* = 5 TRD clinical studies. None of the studies indicated their criteria for choosing aMT or rMT in either cTBS or iTBS stimulation in both healthy participant and patient studies. Importantly, in the selected studies, *n* = 9 used aMT while *n* = 11 used rMT in the healthy participant sample. In the patient sample, *n* = 3 used aMT while *n* = 2 used rMT. More so, the motor threshold (MT) was measured before TBS stimulation and was mostly derived from either the contralateral first dorsal interosseous muscle (Cho et al., 2010) and the right abductor pollicus brevis muscle (Lowe et al., 2014).

In addition, *n* = 20 healthy participant studies adopted the same TBS stimulation procedure as Huang et al. (2005), using a triplet of 50 Hz pulses per train interspersed at 200 ms (5 Hz). However, out of the *n* = 20 studies, *n* = 1 used 900, *n* = 1 used 801, and *n* = 1 used 801 in the cTBS group and 600 pulses *per session* in the iTBS group, whereas *n* = 17 used the mainstream 600 pulses *per session* (Table A2). From our results, it is also observable that a majority of the studies, *n* = 15, used 80% of either aMT/rMT while a few others used different MTs: 90, 75, 57.1, and 50%, *n* = 1 each, and one study used 50, 75, and 100% of aMT/rMT.

Interestingly, almost all the studies that used 80%, *n* = 13, and 90%, *n* = 1, of aMT/rMT recorded a significant TBS stimulation effect either generally or in individual experimental tasks, but 75%, *n* = 1; 57.1%, *n* = 1; and 50%, *n* = 1, either had no or slight stimulation effect. The other study that was conducted using three different MTs (50, 75, 100%) only obtained significant stimulation effect in one of its experiments using 75% RMT.

#### Treatment-Resistant Depression Clinical Studies

Interestingly, there was not much difference between the stimulation intensities used in the clinical and healthy participant studies. However, most clinical studies, *n* = 4, favored 80% of MT with only *n* = 1 using 100% of MT. More so, *n* = 4 of the clinical studies used aMT, and only one used rMT.

When it comes to number of pulses *per session* and stimulation duration, clinical studies at least took twice as long and twice more pulses compared to the healthy participant studies. To be precise, *n* = 4 studies applied at least 1800 iTBS or cTBS pulses *per session*, and those administering a combined iTBS + cTBS applied 3,600 pulses *per session*, 1,800 pulses of each iTBS and cTBS. However, one study applied regular cTBS and iTBS comprising 600 pulses each but used a combined protocol, applying a total of 1,200 pulses *per session*, 600 cTBS pulses to the right DLPFC and 600 iTBS pulses to the left DLPFC (see Table A3 for reference).

## Discussion

This review analyses the effects of cTBS over the DLPFC on human cognition based on systematically selected studies. We have also included iTBS studies to explore its differential effects over the DLPFC compared to cTBS and, thus, facilitate drawing of concrete conclusions. The present review agrees that TMS, including TBS, is a safe and effective method of non-invasively modulating the cortical network when applied according to guidelines and safety procedures (Oberman et al., 2011; Allen et al., 2017). Our analyses also suggest that iTBS stimulation over the left DLPFC have a profound modulating effect on the cognitive domains compared to that to the right. On the other hand, cTBS interfered with cognitive functions when applied to both the left and right DLPFC.

The major question, however, is how can these stimulations be used beneficially rather than to negatively impact cognitive performance? The analyses of this review clearly illustrate that cTBS either over the right or the left DLPFC have mixed effects depending on the cognitive domain targeted.

In decision making, for example, cTBS over the right DLPFC was implicated for positive effects, such as reduced impulsivity in decision making (Cho et al., 2010; Soo et al., 2012). For instance, using a delay discounting task, the above studies find that stimulation over the right DLPFC reduces impulsive decision making by inducing participants to prefer larger delayed rewards to smaller immediate rewards. On the other hand, using model-free and model-based learning algorithms, Smittenaar et al. (2013) demonstrated that cTBS over the right DLPFC negatively influenced decision making by impairing goal-directed behavior. Actually, the dopamine system, which is regulated by nucleus accumbens, has been implicated to play a role in goal-directed behavior (Goto and Grace, 2005; Grace et al., 2007), whereas the stimulation of the DLPFC has been found to influence dopamine release (Hanlon et al., 2015). It is, therefore, possible that cTBS over the DLPFC impaired the interaction between the inputs of the PFC and limbic nucleus accumbens and, thus, affected goal-directed behavior in decision making (Goto and Grace, 2005).

Additionally, cTBS to the right and left DLPFC affected behavior differently, suggesting a hemispheric specialization in learning. In fact, a combined TMS–functional magnetic resonance imaging (fMRI) study using a probabilistic learning task established that cTBS to the right DLPFC increased avoidance-based behavior, whereas that to the left led to the facilitation of reward-motivated behavior (Ott et al., 2011). Arguably, reward-motivated learning is moderated by a complex network formed as a result of PFC–striatum interaction (Torregrossa et al., 2008). Moreover, previous studies report inhibitory stimulation over PFC increased ventro-striatal dopamine release, which is known to elevate reward-related behavior (Frank et al., 2004; Ko et al., 2008; Balleine et al., 2015). Evidently, cTBS to the left and not to the right DLPFC affects the release of dopamine (Ko et al., 2008), and perhaps, this explains the differential hemispheric effect of stimulation in learning.

Cognitive control also decreased after cTBS over the left DLPFC. Essentially, cognitive control monitors and regulates conflict between appropriate and inappropriate information (Kim et al., 2012). Recent studies also implicate left DLPFC in cognitive control, implying that this cognitive function is regulated bilaterally (Gris et al., 2010; Kim et al., 2012). Thus, more research is necessary to establish the effects of bilateral DLPFC-TBS stimulation on cognitive control, especially to investigate whether bilateral iTBS can lead to cognitive control potentiation.

It is also clear that cTBS over the right DLPFC adversely affects attention. cTBS especially to the right DLPFC has been found to depress task-instigated striatal dopamine release, particularly when performing a set-shifting attentional task (Ko et al., 2008). More so, a TMS–EEG study using a tactile discrimination task suggests that cTBS over the right DLPFC reduces the P100 ERP amplitude attentional modulation (Bolton and Staines, 2011). Actually, P100 is speculated to reflect a process of comparing the inbound signals with the existing templates; nevertheless, the functional purpose of P100 is still nebulous (Bolton and Staines, 2011). Therefore, it is arguable that the right DLPFC at least plays a role in attention; however, the hemispherical specialism in attention is still unclear although there is a consensus in the hemispheric asymmetry in some features of attentional control (Aron et al., 2004).

Furthermore, cTBS to left DLPFC led to deterioration in WM performance (Schicktanz et al., 2015). The deterioration of WM performance after cTBS over the left DLPFC suggests that the left DLPFC has a prominent role in WM. However, the cTBS effects were load-dependent, with 0-, 1-, 2-, and 3-back affected differently with medium WM load (2-back) performance mainly affected (Schicktanz et al., 2015; Vékony et al., 2018). Basically, it is expected that an increase in working memory load leads to more activation and lack of effect on performance after cTBS may be due to the performance compensation by the vast activation network or, perhaps, other factors such as the method used to identify coordinates even though, in another study, iTBS led to improvement in a 3-back task (Chung et al., 2018b).

In language, EEG and behavioral results based on a non-verbal switching task demonstrate that neither iTBS nor cTBS over the left DLPFC affects the language switch control. This suggests that, despite acting as a mechanism for language control (Hagoort, 2015), the left DLPFC does not regulate LS (Pestalozzi et al., 2020). However, the lack of cTBS effects on LS may be due to the possibility of recruitment of bilateral resources and/or the large neural network involved in LS (Hosoda et al., 2012). Therefore, interfering with one focal brain area may not significantly alter the switching cost. On the other hand, the electrophysiological results showing the stimulation effect confirm the role of the left DLPFC in top-down control of task demand maintenance (Macdonald et al., 2000). It is also interesting to note the interaction between different cognitive domains, such as working memory and cognitive control, in language control (Luk et al., 2012; Klaus and Schutter, 2018), where the working memory keeps online the relevant language information.

Moreover, it is evident that the left DLPFC modulates speech. The cTBS over the left DLPFC not only led to increased vocal compensation for pitch perturbation, but was also responsible for the reduction in cortical responses (Liu et al., 2020). This implies that the left DLPFC exerts a top-down inhibitory control on vocal production in response to auditory feedback, which was diminished after cTBS stimulation.

Generally, cTBS had an inhibitory effect on the cognitive performance in most of the healthy participant studies except in a few domains, such as planning, where it increased the speed of planning decision making; this is in line with other TMS studies (Huang et al., 2005; Gentner et al., 2008; Gamboa et al., 2010; Verbruggen et al., 2010; Suppa et al., 2016). The improvement of planning after cTBS to the left DLPFC can be explained by the analogy of facilitation through “addition by subtraction” suggested by Luber and Lisanby (2013, p. 3). These researchers argue that TMS can cause cognitive facilitation by disrupting other processes that either compete with the main task for the limited cognitive resources or distract task execution. Conversely, iTBS, especially over the DLPFC, resulted in amelioration of performance although there was no cognitive effect in two iTBS studies. It is, therefore, imperative to understand the dynamic effect of TBS stimulation on diverse cognitive domains when applying it in neuro-psychiatric intervention.

One of the important factors that could explain the differential effect of TBS in the included studies is the varying methods of locating the stimulation area(s). In fact, a majority of the studies, *n* = 14, did not use neuronavigation, and this could have contributed to the inconsistencies in the DLPFC TBS cognitive effects (Table A1). Evidently, the use of neuronavigation increases the accuracy of locating the exact brain area targets to be stimulated (Lefaucheur, 2010; Lage et al., 2016). In addition, Vaghefi et al. (2015) suggest that, apart from eliminating the error stemming from manual positioning of the coil, neuronavigation also makes it possible to gauge the position of the coil in real time and to approximate the flow of induced current. Essentially, neuronavigation is made possible by integrating MRI, fMRI, or PET brain imaging information (Lefaucheur, 2010).

However, most studies are still using the EEG 10-20 system that is reliant on the skull's ordinary landmarks (Klem et al., 1999; Herwig et al., 2003). In our review, especially, a significant number of studies used F3 and F4 positions to locate, the left and right DLPFC, respectively, and one study used the F1 position for the left prefrontal cortex. This is despite the inaccuracies that can be realized as result of heterogeneous morphology between individuals (Rusjan et al., 2010). Therefore, it is highly recommended that future research should use neuronavigation to precisely place the coil to stimulate the target brain area and consequently boost the accuracy and robustness of the stimulation effects.

Furthermore, the control experiment is crucial in distinguishing the actual modulatory effect arising specifically from TBS stimulation and that associated with sensory and psychological confounds (Duecker and Sack, 2015; Jung et al., 2016). In the present study, *n* = 20 studies used either sham or vertex stimulation, and *n* = 1 used neither; instead it utilized baseline (pre-stimulation) measures for comparisons. Nevertheless, different sham stimulation was evident in various studies (Tables A2, A3), and this standardization deficit in the sham procedure or the lack of it in some studies makes it difficult to succinctly compare the cognitive effects in diverse studies.

Apart from the use of sham to control for the unintended effects, blinding (camouflaging the allocation of group from one or more research stakeholders) also minimizes the possible bias of performance and/or that of assessment in an experiment depending on the blinding approach adopted (Karanicolas et al., 2010). A majority of the healthy participant studies used single blinding, *n* = 17, whereas all the patient studies used double blind (blinding of both participants and the researchers), and two studies did not use blinding at all (Table A1). We, therefore, suggest that future studies, in addition to using pseudorandomization, should also choose the most appropriate blinding procedure. According to Day and Altman (2000), the decision of choosing the kind of blinding to use depends especially on the study characteristics; for instance, characteristics, such as calibration of pain using scoring scales, is prone to be subjective while quantitative features, such as number of remissions, can be quantitatively and objectively ascertained.

Again, our review observes that some of the TBS modulatory effect may not lead to significant behavioral moderation, and such changes may only be captured through other non-behavioral complementary techniques, such as EEG, PET, and fMRI. Previous TMS studies have demonstrated that it is possible to use imaging and signal detection techniques alongside behavioral tasks, such as *n*-back (Ko et al., 2008; Hautzel et al., 2009; Ott et al., 2011; Hoy et al., 2015; Kaarre et al., 2018). The use of multimodal techniques augments the results and makes it possible to have a vivid understanding of the behavioral and physiological impact of TBS on a specific brain region.

Much more, it is evident that the type of behavioral task and the specific time conducted after TBS stimulation can influence the results. Particularly, one study used clinical tasks to test WM and EF in healthy participants, and generally, there was no significant stimulation effect noted (Viejo-sobera et al., 2017). It could be interpreted that the use of clinical tests in healthy participants is counterproductive. However, such results may be explained by other determinants, including the stimulation type and intensity. Thus, more comparative studies are necessary to assess the effectiveness of clinical tests on healthy subjects. It is also noteworthy that the inhibitory effect of cTBS 600 pulses is weaker compared to the facilitatory effect of similar iTBS 600. According to Wischnewski and Schutter (2015), the iTBS potentiation lasts up to 60 min, whereas the cTBS inhibition lasts for 50 min, and the variation in effects between the two is greater within 30 min after stimulation. For this reason, cognitive tasks and measurements should be done within the period when the TBS effects are still present. The greater TBS potentiation is consistent with other rTMS paradigms, which have showed the same trend of a more facilitatory than depressing effect (Maeda et al., 2000; Gorsler et al., 2003; Houdayer et al., 2008; Rajji et al., 2011; Delvendahl et al., 2012).

### Optimum Stimulation Intensity

**Figure 2:**
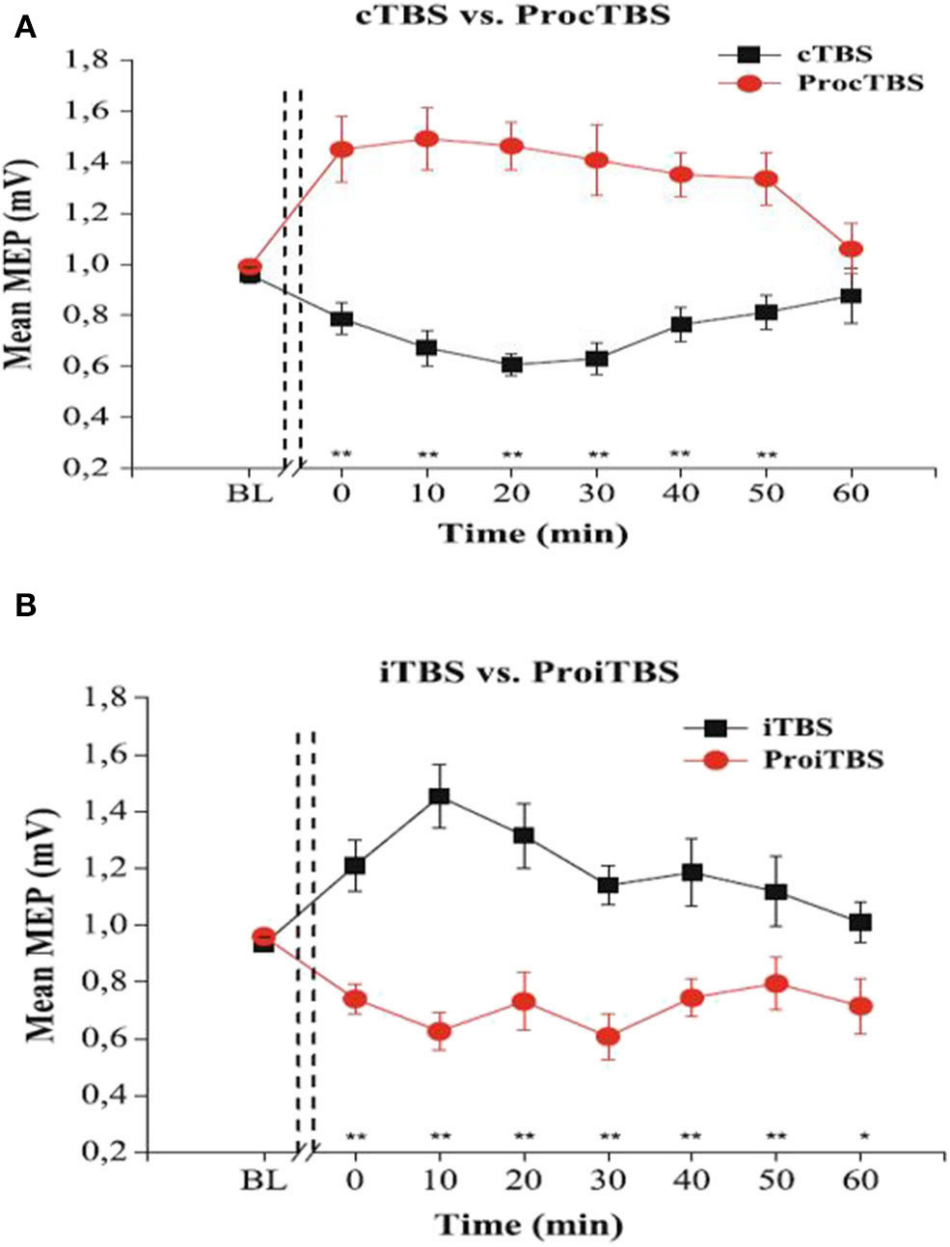
The effects of TBS and Prolonged TBS on the motor evoked amplitude mean, extracted with permission from Gamboa et al. (2010). **(A)** showed an inverted (Potentiated) MEP amplitude after application of prolonged cTBS compared to ordinary cTBS_600_ (inhibited). **(B)** shows inverted (Inhibited) MEP amplitude after prolonged iTBS compared to ordinary iTBS_600_ (potentiated). Error bars indicate standard error (**P < 0.01).

The amount of TBS stimulation to apply *per session* is of great interest to neuroscience research. Indeed, the amount of stimulation is determined by the intensity and the number of pulses *per session* (Tables A2, A3). Generally, it is conceivable that an increase in intensity should lead to better performance, but interestingly, overly increasing the stimulation duration or intensity are found to reverse the effects in healthy participants (Gamboa et al., 2010). Indeed, these authors demonstrate that prolonged iTBS and cTBS causes inverted MEP effects, depression, and potentiation, respectively, compared to the ordinary iTBS600 or cTBS600. They illustrated the inverted effects as seen in (Figure 2).

Furthermore, previous studies have established that cTBS applied for as short as 20 s modulates the corticospinal networks. However, there is a lack of agreement on the direction of cTBS300 Huang et al. (2005) on one hand suggesting that it causes depression but only for 20 min compared to 60 min achieved by cTBS600 and Gentner et al. (2008) on the other hand, proposing that cTBS300 causes corticospinal inhibition only when preceded by voluntary muscle contraction, otherwise it leads to corticospinal excitation. The findings that very limited time (20 s) cTBS can modify the neural network is a step forward toward understanding the mechanism of neural moderation although additional research is required to establish the precise regulatory effects of cTBS300 on the corticospinal system.

In addition, we observe that the stimulation intensity range is the same for patient and healthy participants with the lowest intensity being 50% and the highest being 100% of the MT (Tables A1, A2). However, a bigger number of included studies used 80% MT despite MT not being our exclusion criteria, and it is also fascinating that significant stimulation effects were reported in the majority of these studies (Tables A1, A2). More so, the application of uniform intensity for all participants in most studies despite the peculiar individual MT differences is in line with previous findings (Kaminski et al., 2011).

Moreover, the efficacy of the 80% MT stimulation confirms the findings of previous work, which demonstrated that 75% and not 50% or 100% MT produces the strongest stimulation effect (Chung et al., 2018a) even though the results reported in the included studies could have also been influenced by other factors, including errors arising from manually localizing the stimulation site in the studies that did not use neuronavigation. Importantly, similar MT threshold parameters were used for both iTBS and cTBS.

Finally, the present study establishes that perhaps more TBS stimulation is required in treatment of TRD patients than is required in healthy participants for its effect to be realized. The number of pulses *per session* in the included patient studies ranged from 1,200 to 3,600, whereas in the healthy participant studies, it ranged from 600–1,200 (Tables A2, A3). It may be interpreted that the MDD patients tend to have greater tolerance to greater stimulation compared to the healthy participants.

### Cross-Frequency Coupling in Depression

**Figure 3:**
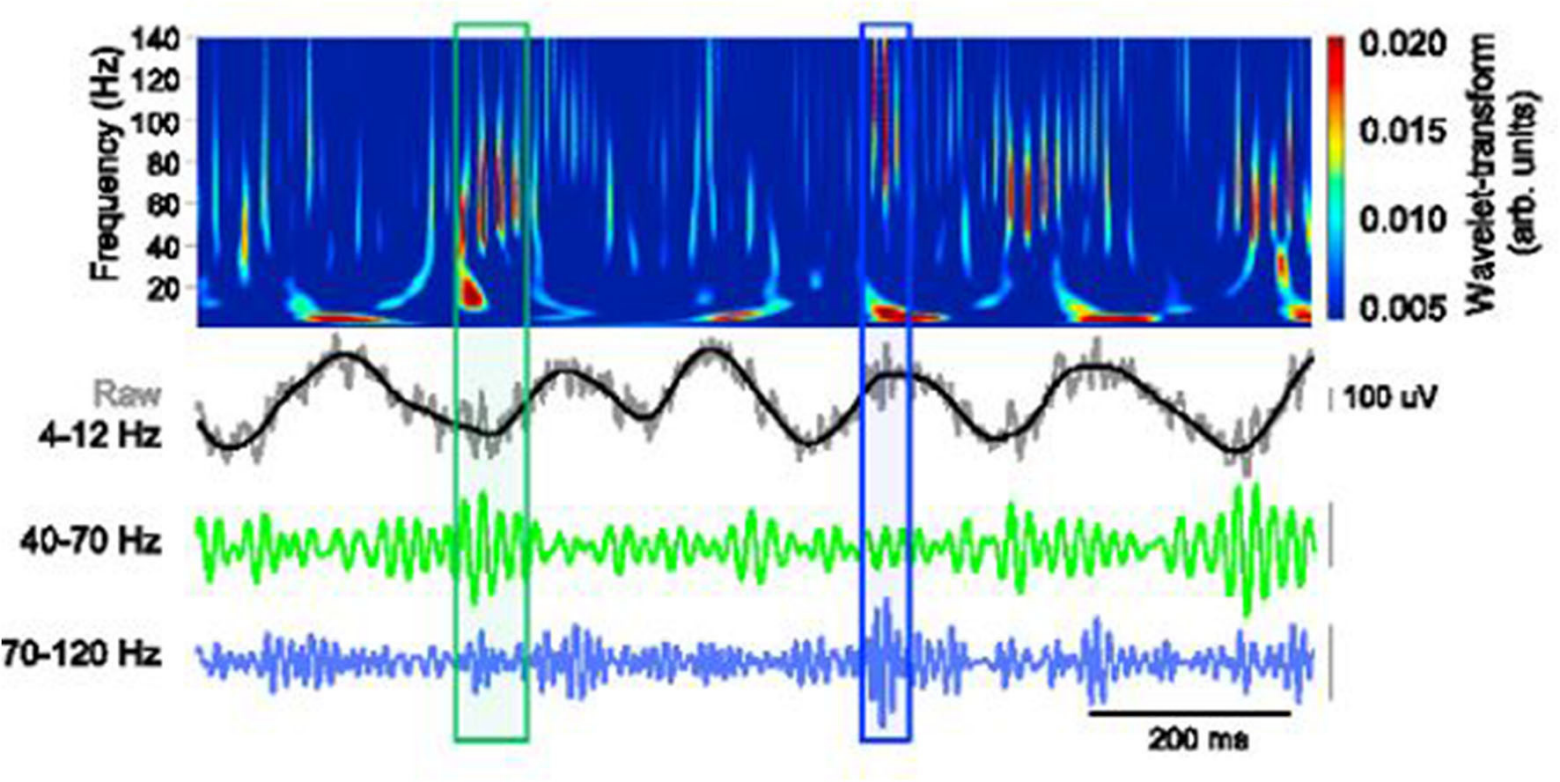
Schematic depiction of theta-gamma coupling. Lower traces, slow (40–70 Hz, green) and fast (70–120 Hz, blue) gamma events, occurring at distinct phases of the theta oscillation (black). Obtained with permission from Stujenske et al. (2014).

Findings from recent studies emphasize the importance of theta oscillations and gamma rhythms in cognitive function. More specifically, theta–gamma coupling has been established to be vital in neural plasticity and communication (Axmacher et al., 2010; Canolty and Knight, 2012; Palva and Palva, 2018). The cross-frequency coupling (CFC) as captured in a working memory recall task is clearly demonstrated in Figure 3.

The neuronal CFC may, however, be disoriented in patients with psychiatric conditions, including depressive disorder, and this could explain the inherent cognitive function deterioration in these patients (Adhikari et al., 2010; Zheng and Zhang, 2015). Clearly, in their review, Fitzgerald and Watson (2018) suggest that gamma rhythms differ from healthy controls and major depression patients and also observed that depression-like behavior in animal studies was associated with atypical gamma rhythms. This highlights the significance of gamma rhythms whose coupling with theta is crucial for cognitive functions.

Additionally, brain imaging MDD studies have mapped out a supposed depression brain network. Notably, this network involves the limbic, hippocampus and amygdala; cortical brain areas; frontal, dorsal anterior cingulate cortex and ventral; and nucleus accumbens (Mayberg, 2009; Smart et al., 2015). One of the neural pathways that has been targeted for the treatment of depression is the ventral hippocampus–medial prefrontal cortex pathway (mPFC) (Carreno et al., 2016). In their animal model, Zheng and Zhang (2015) propose that depression weakened the one-way CA1 → mPFC theta phase coupling; however, local field potential induction led to CFC enhancement. It should be noted that CA1 neurons in the hippocampus are responsible for episodic memory formation, consolidation, and retrieval (Bartsch et al., 2011).

Furthermore, TMS over the left DLPFC selectively decreases subgenual anterior cingulate cortex (sgACC) hyperconnectivity, regulates the default mode network (DMN)–central executive network (CEN) interaction (Liston et al., 2014; Smart et al., 2015), and increases gamma activity in the PFC (Kito et al., 2014). Arguably, depression is associated with atypical DMN connections, which is possibly due to emotional regulation shortfalls (Liston et al., 2014), and TMS long-term potentiation is expected to improve these connections. In fact, high-frequency TMS has been found to induce the network within the DMN areas between DLPFC and mPFC (Liston et al., 2014).

Therefore, we can argue that TBS modulates the CFC by either enhancing it (iTBS) or inhibiting it (cTBS). It has been demonstrated that excitatory TMS applied over the left PFC in depression patients enhances theta–gamma coupling and has been associated with improvement in depressive symptoms (Noda et al., 2017).

### Application of cTBS Over DLPFC in Treatment of Pharmacotherapy Refractory Major Depression

The implication of the DLPFC in major depression (Lemogne et al., 2010; Sibille et al., 2011) has made it a key target in treatment intervention. It is also important to note that the DLPFC has been strongly associated with basic executive functions, such as working memory and attentional and inhibitory control as well as higher level EF processes, such as planning and intelligence (fluid intelligence) (Miller and Cohen, 2001; Wood and Grafman, 2003; Forbes et al., 2014; Snyder, 2014; Caspers et al., 2017). This implies that there is a close interlink between the dysfunction of executive function and major depression, and it is also possible that MDD is a product of a serious cognitive breakdown. This is affirmed by Li et al. (2014), who establish that many patients with pharmacotherapy-resistant depression had deteriorated cognitive function, specifically the EF.

The current study includes five TRD clinical studies in the review to find out the effectiveness of cTBS in treatment intervention. Indeed, cTBS inhibits cortical neural activity, which generally modulates the entire related cortical network (Huang et al., 2005; Galea et al., 2009; Schicktanz et al., 2015; Chen et al., 2016). The present review establishes that cTBS, especially to the right hemisphere, is able to influence MDD patients' response to medication. Three out of five patient studies included in our review indicate that cTBS to the right DLPFC caused a modest response to medication (Li et al., 2014; Chistyakov et al., 2015; Cheng et al., 2016). However, one of them indicates a deterioration in the executive function after cTBS to the right DLPFC and no EF after iTBS to the left DLPFC (Cheng et al., 2016). The paradoxical deterioration of the EF after cTBS stimulation, even though there was a trend toward response to medication, can be explained by the possibility of the right DLPFC cTBS inhibitory effects being transmissible to the other brain regions of the neural cortex causing global inhibition (Gratton et al., 2013) or the ability of the Hamilton Depression Rating Scales (HDRS) to measure other improvements that may not be necessarily directly linked to EF (Hamilton, 1960), and this may explain why cognitive impairment often persists beyond remission (Lam et al., 2014; Cheng et al., 2016). One of the clinical studies only used a combined treatment, cTBS + iTBS, so it was not possible to deduce the singular effects of cTBS in the study (Plewnia et al., 2014).

Furthermore, the iTBS to the left DLPFC and cTBS + iTBS to the right and left DLPFC, respectively, led to a greater improvement (reduction) in HDRS scores. However, it's only in iTBS and not cTBS + iTBS that there was a significant EF enhancement. The latter had no effect on EF. This is in line with earlier healthy participant studies, which demonstrate that the facilitatory rTMS over the left DLPFC could result in non-mood-dependent EF amelioration (Moser et al., 2002). A plausible explanation for the lack of EF effect by cTBS + iTBS is the possibility of the cTBS neutralizing the potentiation effect of iTBS (Chung et al., 2018a).

Arguably, the application of inhibitory stimulation (cTBS) over the right DLPFC and facilitatory (iTBS) over the left DLPFC could have been informed by the proposition that MDD is associated with hypoactivity and hyperactivity to the left and right PFC, respectively (Li et al., 2014). This notwithstanding, the tendency of iTBS over the left DLPFC to bear more antidepressant effect compared to the modest effects after the right DLPFC is contrary to the findings of Fitzgerald et al. (2003), who demonstrate that higher frequency rTMS (HFT) and lower frequency rTMS (LFT) had the same antidepressant effect. Such discrepancy is intriguing bearing that HFT has a similar potentiation effect as iTBS and LFT has a similar inhibitory effect as cTBS (Lowe et al., 2018).

Nevertheless, the combined stimulation approach (cTBS + iTBS) demonstrates the ability of the cTBS to be integrated with other protocols for treatment intervention. The amalgamation of cTBS + iTBS in *n* = 4 patient studies led to the best response to medication with *n* = 1 resulting in 31% patient symptomatic remission compared to singular cTBS or iTBS (Li et al., 2014; Plewnia et al., 2014; Cheng et al., 2016; Dhami et al., 2019). Despite the fact that this combination seems to provide a supplement to medication-resistant MDD, more research is required to rule out the effect of increased stimulation session duration and number of pulses *per session* that may come as a result. Additionally, healthy participant studies are also necessary to establish the cognitive effects of the combined cTBS and iTBS stimulation.

Moreover, the fact that there was no effect of stimulation intensity difference as *n* = 4 of the patient studies used 80% aMT or rMT and *n* =1 used 100%. This suggests the effectiveness of similar TBS stimulation intensity for both patient and healthy subjects and supports the findings of earlier studies that demonstrated the optimum stimulation intensity is 75%, and thus, increasing or reducing it significantly may affect the results negatively (Chung et al., 2018a; Vékony et al., 2018).

Finally, despite TBS providing a promising compliment to medication in the treatment of pharmacotherapy-resistant depression, its long-term efficacy in the post-treatment period is still less documented. It is possible that TBS's beneficial effects in the treatment of refractory MDD may fade over time just like has been observed in rTMS treatment (De Raedt et al., 2015). More research is, therefore, required to ascertain the long-term benefits of TBS in treatment of medication-resistant depression after remission.

## Conclusion

Evidently, the DLPFC plays a crucial role in cognition with numerous cognitive domains impacted when TBS is applied over this region. Clearly, in the included studies, cTBS over the DLPFC caused mixed effects on cognitive processes depending on the hemispheric target of the stimulation. Specifically, cTBS to the right DLPFC impaired inhibitory control, planning, and attention and had mixed effects with impaired goal-directed behavior on one hand and reduced impulsivity on the other. More so, cTBS over the left DLPFC negatively affected executive function, working memory, language (affected vocal production), and cognitive control and also conversely led to faster planning. Inherently, the cTBS is expected to inhibit cognitive functions; however, the notable ameliorating effect can be explained by the phenomenon of “addition by subtraction,” in which the competing cognitive processes and/or distractors are inhibited by the cTBS application and, thus, enhanced performance (Luber and Lisanby, 2013). On the other hand, iTBS especially over the left DLPFC improved working memory performance but not decision making.

Taken together, cTBS provides a promising approach of neural modulation due to its safety and short period of application. These results should, however, be read with caution as a small sample of studies was used and some the included studies did not use neuronavigation while others lacked experimental controls.

## Data Availability Statement

All datasets generated for this study are included in the article/Supplementary Material.

## Author Contributions

RN and JZho conceived the idea and drafted the manuscript. RN, LL, JZha, and ZJ carried out the literature search and data extraction. All data analyses were performed by RN and LL. All authors reviewed the manuscript.

## Conflict of Interest

The authors declare that the research was conducted in the absence of any commercial or financial relationships that could be construed as a potential conflict of interest.
