# Supplementary material for: Assessing the Effects of Continuous Theta Burst Stimulation Over the Dorsolateral Prefrontal Cortex on Human Cognition: A Systematic Review
Source: Front Integr Neurosci. 2020 Aug 4;14:35. doi: 10.3389/fnint.2020.00035 (PMC7417340; doi:10.3389/fnint.2020.00035)
Supplement: Supplementary file 1 [file Table_1.docx]

Appendices

****Appendix 1: Table A1****

**Table 1: Illustration of the Studies**

**Studies (H)Healthy/ No Single Double- Within Between- Mixed**

**First author (P)Patient Blinding Blind Blind Subject Subject Design**

Li, (2014), Plewnia, (2014), Chistyakov, (2015), Cheng, (2016) P X X

(Dhami et al., 2019) P X X

Ko, (2008), Cho, (2010), Soo, (2012), Smittenaar, (2013), H X X

Georgiev, (2016), Mcneill, (2018) and Hoy, (2015),

(Chung , 2017) and (Liu et al., 2020)

Ott, (2011), Bolton, (2011), Schicktanz, (2015), H X X

(Vékony, 2018) and Viejo-sobera, (2017)

Lowe, (2014) Maier, (2018) H X X

(Kaller, 2011), (Pestalozzi et al., 2020) and H X X

(Langenbach et al., 2019)

Wook, (2018) H X X

****Appendix 2: Table A2****

**Table 2: TBS over DLPFC and Cognition in Healthy Participants**

**First Author No. of NN aMT/ Percentage Stimulation Pulses per Session Hemisphere sham/ Measurement Cognitive Cognitive Effects**

**Subjects rMT Threshold Type Session Duration control site Technique Domain**

(Ko, 2008) 10 Yes aMT 80 cTBS 900 40s L Vertex MCST EF Impaired EF performance

Impaired dopamine release

R No effect on EF performance

(Cho, 2010) 7 Yes aMT 80 iTBS 600 192s R coil at 90⁰ DD, BDI Decision Making No effect on impulsive decision making

to the target

cTBS 600 40s R DD, BDI Decreased impulsivity

(Ott, 2011) 47 Yes aMT 80 cTBS 600 40s R Vertex Probabilistic Learning Enhanced reward sensitivity and striatal

Learning task prediction

Yes aMT cTBS 600 40s L increased avoidance based behaviour

(Bolton, 2011) 14 No rMT 80 cTBS 600 40s R Current directed Tactile Attention Reduced attention-dependent regulation

Up and outward DT of somatosensory ERP

(stimulator output 6%)

(Kaller, 2011) 52 Yes rMT 80 cTBS 600 40s R Over PPC and Tower of Planning Deceleration in cognitive processing

coil tilted 90⁰ London (slower planning)

away from scalp

Yes rMT cTBS 600 40s L Accelerated processing speed

(faster planning)

(Soo, 2012) 8 Yes aMT 80 cTBS 600 40s R coil at 90⁰ DD, MD, PD Decision making Reduced impulsivity

(Smittenaar, 2013) 25 No aMT 90 cTBS 600 40s R Vertex MB-MF hybrid Decision making Impaired goal-directed behaviour (GDB)

Impaired probabilistic learning

No aMT 90 cTBS 600 40s L Impaired GDB only in low WM

(Lowe., 2014) 21 No rMT 80 cTBS 600 40s L coil 90⁰ to stroop, EF Deterioration of EF

Head surface go/no-go (increased food craving and a selective

Stop signal task uptake of high-calorie food)

(Schicktanz, 2015) 40 Yes rMT 80 cTBS 600 40s L Sham N-back, STAI, WM Decreased accuracy only in 2-back task

(not specified) VAS

(Hoy, 2015) 19 No rMT 80 iTBS 600 190s L 90⁰ coil N-back WM General improvement in wm performance

Rotation about the Trend towards improvement in RT

handle’s axis

(Georgiev, 2016) 15 No aMT 80 cTBS 600 40s R 20% AMT and MDT Decision making Impaired decision making in easy trials

90⁰ coil tilt from surface

(Viejo-sobera, 2017) 36 Yes aMT 57.4 cTBS 600 40s L coil 90⁰ to scalp N-back, stroop, WM, EF Generally, no significant stimulation effect

surface Digit backward, at individual level, cTBS had effect on some

Tower of Hanoi tasks performance

Yes aMT 57.4 iTBS 600 192s L Generally no significant stimulation effect

Significant effect of iTBS in some individual tasks

(Chung, 2017) 16 No rMT 50,75,100 iTBS 600 192s L N/A N-back WM Improved wm performance only 75% 3-back

(Maier, 2018) 19 No rMT 80 cTBS 600 40s R Active to Passive UG, DG Decision making Deterioration of forgiveness decision making

placebo Cognitive control Increased revenge behaviour/ decrease control

(Wook, 2018) 18 No rMT 75 iTBS 600 192s L 90⁰ coil rotation N-back WM No improvement in WM performance

right about

Handle’s axis

No rMT 75 iTBS + iTBS 1200 384s L N-Back WM No improvement in WM performance

(Mcneill, 2018) 20 No rMT 80 cTBS 600 40s R Vertex Bogus taste test Inhibitory Reduced inhibitory control and consequently

SST, BIS-11 control increased alcohol intake

AUDIT

(Vékony, 2018) 51 Yes rMT 50 cTBS 600 40s R/L 45⁰ coil N-back WM Generally, no WM effect on both hemispheres

Rotation away from Slight behavioural effect (Inhibition of practice

Skull, handle pointing effect)

backward

Yes rMT 50 iTBS 600 190s R/L N-back WM No behavioural effect

(Langenbach et al., 2019)

93 Yes rMT 80 cTBS 801 44s R Vertex control Fishing Game Decision No stimulation effect on decision making pertaining

and sham coil making future generation

(Liu et al., 2020) 21 No aMT 80 cTBS 600 40s L Coil rotated at 90⁰ FAF-based Language Enhanced vocal compensation for pitch perturbations

to skull surface in vocal production Reduced P2 cortical responses

sham

(Pestalozzi et al., 2020)

41 No aMT 80 iTBS 600 190 L Sham coil Picture naming Language No difference between iTBS and cTBS results

No aMT *0 cTBS 801 44s L Non-verbal Compared to sham, iTBS subjects were faster

Switching task EEG analyses found stimulation effect in picture naming

No interaction effect between stimulation and block

(switching versus non-switching)

Note: AMT = Active Motor Threshold, AUDIT= Alcohol Use Disorder Identification Test, BDI = Beck’s Depression Inventory, BIS-11 = Barret Impulsivity Scale, DD = Delay Discounting, DG = Dictator game, DT = Discrimination task, EEG = Electroencephalogram, EF= Executive function, FGQ-S = Food Craving Questionnaire-State, GDB = Goal-Directed Behaviour, H = Healthy, L = Left, MB = Model Based, MF = Model Free, MCST = Montreal-Card-Sorting-Task, MDT = Moving Dot Task, MD = Magnitude discrimination, NN = Neuronavigation, PD = Physical Discrimination, PPC = Posterior Parietal Cortex, R = Right, RMT = Resting Motor Threshold, STAI = Spielberger State Anxiety Inventory, SST = Stop Signal Task, UG = Ultimatum Game, VAS = Visual Analogue Scale and WM = Working Memory.

****Appendix 3: Table A3****

**Table 3: Effects of TBS on Major Depression Medication-Treatment Response**

**First Author Participant NN AMT/RMT % Threshold Stimulation Pulses per Session Hemisphere Sham/ control site Measurement Stimulation Effect**

**Type session Duration Technique**

(Li, 2014) 60 No aMT 80 cTBS 1800 120s R Coil at 90$⁰$ against DSM-IV, MINI, Slight improvement; 25% of subjects

the skull CGI-S, HDRS, MSM responded after 2 weeks

iTBS 1800 570s L Significant improvement; 40% response rate

Better antidepressant effect than cTBS and sham

cTBS + iTBS 3600 690s R and L The best response rate of 66.7% after 2 weeks

Generally, had highest antidepressant effect

(Plewnia, 2014) 16 No rMT 80 cTBS + iTBS 1200 40s/190s R and L 45⁰, 5 cm Lateral BDI, Significant response to MD medication

to F3 & F4 HAMD 31% of patients indicated remission

(Chistyakov, 2015) 29 No aMT 100 cTBS 3600 49 mins R Specially designed DSM-IV, HDRS Generally modest stimulation effect Sham coil with same Marked improvement after 2 weeks (33.3%) Sound but no stimulus sensation Greater improvement after 4 weeks (60%)

response rate. No sign. difference from sham.

(Cheng, 2016) 60 Yes rMT 80 cTBS 1800 120s R coil at 90⁰ to the DSM-IV, HDRS-17, Trend towards worsening EF

Skull MINI, CGI-S, WCST Slight response to medication

iTBS 1800 570s L Amelioration of the executive function

iTBS + cTBS 3600 690s L and R Had best antidepressant effectiveness

no effect on the executive function

(Dhami et al., 2019) 20 Yes aMT 80 iTBS + cTBS 3600 690s L and R N/A BDI, HRSD-17 General improvement in depressive symptoms

(sign reduction in HRSD-17 scores vs BL)

4 patients responded and 2 of them achieved remission

Note: CGL-S = Clinical Global Impression Scale, DSM-IV = Diagnostic Statistical Manual Fourth Edition, HDRS = Hamilton Depression Rating Scale, HRSD = Hamilton rating Scales for Depression -17 (Original Version), MINI = Mini International Psychiatric Interview, MSM = Maudsley Staging Method and WCST = Wisconsin Card Sorting Test.
